# 

**Published:** 1857-01

**Authors:** 


The Readers of the *Journal of Public Health and Sanitary Review,* are respectfully informed, that with the first number of the new volume, due April 1857, the title of the Journal will be

### THE SANITARY REVIEW

AND

### JOURNAL OF PUBLIC HEALTH.

The reason for this slight modification is explained in the Correspondents' column of the present number.

---

**To New Subscribers to the Journal of Public Health.**

The Publisher begs to intimate, that new Subscribers wishing to have the series of the Journal complete from the first number, can obtain sets of Vol. I, at Subscribers' prices, 10s. post free, by application to him, enclosing Post-office Order, payable at the Charing Cross Branch to Thomas Richards.

The Journal of Public Health is sold by all Booksellers, and is equally suited for the general and professional reader, for the scientific society, and for the private library.

London: T. RICHARDS, 37, GT. QUEEN STREET.

---

Subscribers who have not yet paid their subscriptions to the present volume, are requested to forward them by Post-Office Order, or otherwise, to T. Richards, 37, Gt. Queen Street, London, W. C. Orders should be made payable at the Charing Cross Branch.

---

MICROSCOPICAL & ANATOMICAL DRAWINGS of all kinds are executed from nature, for the Medical Profession, Naturalists, and others, with strictest care as to details and artistic perfection, and at moderate cost, by Mr. LENS ALDOUS, 3 Keppel Row, Stoke Newington Green, artist of numerous works for Royal College of Surgeons and other Scientific Institutions.

### NOTES AND CORRESPONDENCE.

The Editor is anxious to give free scope to the expression of opinion› but does not hold himself responsible for the opinions of those of his contributors who attach their names to the articles furnished by them.

The title of the Journal will be slightly modified with the new Volume. The brief part of the title, "SANITARY REVIEW," will be placed first. This change is made to prevent a mistake that has once or twice occurred, in confounding this work with a production called Health Journal, or some such name, which occasionally is issued, but the objects and views of which are of a totally different character to the views and objects of those who contribute to these pages.

The classification of diseases adopted by Dr. Farr is known to us. The term zymotic is well understood, but we agree with our Correspondent that the other classifications, viz., "Cachectici," "Monorganici," "Metamorphici" and "Thanatici," are so many mistakes, which it were better not to adopt. Such classification would lead to great confusion, and to the further unnecessary complication of disease nomenclature. At the same time it must remembered that Dr. Farr has great difficulties to meet in classifying his returns. What is required most, is an entirely new nosology based on scientific principles, simple in its general form and brief in its particulars. There are not many elementary forms of disease.

### LOCAL OBSERVERS.

The reports of our Local Observers will in time be very valuable records. We have now the whole length of Great Britain under observation, and nearly fifty earnest men engaged in the work. Such an army of observers, all working simultaneously in recording the diseases before them, has certainly never before been in action, and it is one of the cheering prospects connected with the supervision of this Review, to feel how ably and steadily it is thus supported.

In commencing Volume III, we are anxious to increase the number of local epidemiologists, and in order that their reports may be still more valuable, we suggest that in the additional observations supplied, facts may be given as far as possible on the following points.

1. On the *origin* of any epidemic which may have been prevalent. Was the epidemic traceable to an imported case, or did it arise spontaneously?

2. On the effects of an epidemic as modifying other prevailing epidemics, or as running side by side with others.

3. On the external causes which have in any special epidemic favoured the development of the disease.

4. On any causes which may seem to have led to the arrest of an epidemic disease.

5. Whether the distinct types of an epidemic, specially of scarlet fever, are due to the mere propagation of the type from one person to another; or whether it is the general disease which is propagated, the type being the result of external influences only, such as impurity of air or water, or some atmospheric peculiarity.

Lastly, the Editor is anxious to obtain some absolute facts in reference to ozone, and its influences in relation to disease. He will, therefore, to such of the Local Observers as will undertake the task, forward regularly with the report sheet, a packet of Moffat's ozone papers with directions as to use. If fifty Gentlemen were all at the same time engaged in observing the ozone paper, and connecting with such observations those relating to the presence and prevalence of diseases, the vexed question now so much discussed, as to the effects arising from the presence or absence of ozone on different forms of disease, would soon be set at rest.

### BOOKS RECEIVED.

BALLARD (Edward, M.D.) First Quarterly Report of the Medical Officer of Health for Islington. London: 1856.

BARNES (Robert, M.D.) Second Quarterly Report on the Sanitary Condition of Shoreditch, for quarter ending September 27th, 1856. London: 1856.

DRUITT (Robert, L.R.C.P.) Report on the Quality of the Waters used in the in-Wards of the Parish of St. George's, Hanover Square. London: 1856.

GAMGEE (Joseph Sampson) Researches in Pathological Anatomy and Clinical Surgery. London: Baillière. 1856.

LAYCOCK (Thomas, M.D.) On the Pathology and Treatment of the Contagious Furunculoid. Edinburgh: 1856.

LEARED (Arthur, M.D.) Analysis of 136 Cases of Phthisis. Reprint from Medical Times. 1856.

LETHEBY (Henry, M.B.) Report on the Sanitary Condition of the City of London for the years 1855-56. London: 1856.

MARCET (W., M.D.) The Composition of Food, and how it is Adulterated. London: Churchill. 1856.

McDOUGALL (Alexander.) On the Preservation of Natural Manures, London: Whittaker & Co. 1856.

REGISTRAR GENERAL'S Seventeenth Annual Report. London: 1856.

ROUTH (C. H. F., M.D.) Fæcal Fermentation as a Cause of Disease. London: Richards. 1856.

STARK (James, M.D.) Report on the Meteorology of Scotland. Edinburgh: Murray and Gibb. 1856.

SALTER (Hyde, M.D.) An Introductory Address delivered at Charing Cross Hospital. London: 1856

THOLOZAN (Dr.) Recherches sur les Maladies de l'Armée d'Orient pendant l'Hiver de 1854-1855. Paris: 1856.

(An admirable paper, full of that clear common sense and extended knowledge which mark all the scientific productions of Dr. Tholozan.)

TILT (E. J., M D.) The Change of Life in Health and Disease. London: Churchill. 1856.

WESTALL (EDWARD.) Quarterly Report on the Mortality of Croydon. To September 1856.

WILLIAMS (James, M.D.) The Topography and Climate of Aspley Guise. London: Richards, 1856.

---

### IN EXCHANGE.

Literary Gazette.
Psychological Journal.
Association Medical Journal.
Ranking and Radcliffe's Abstract of the Medical Sciences.
Chemist.
Veterinarian.
Asylum Journal of Mental Science.
Quarterly Journal of the Chemical Society.
Scottish Review.
Edinburgh Medical Journal.
Glasgow Medical Journal.
Dublin Hospital Gazette.
American Journal of the Medical Sciences.
New York Journal of Medicine.
Montreal Medical Chronicle.
Charleston Medical Journal.
Gazette Médicale de Paris.
Aerzliches Intelligenz-Blatt.

